# Assessment of Predictive Markers for Placental Inflammatory Response in Preterm Births

**DOI:** 10.1371/journal.pone.0107880

**Published:** 2014-10-07

**Authors:** Min-A Kim, You Sun Lee, Kyung Seo

**Affiliations:** Department of Obstetrics and Gynecology, Gangnam Severance Hospital, Institute of Women’s Life Medical Science, Yonsei University College of Medicine, Seoul, Korea; University of Cincinnati, United States of America

## Abstract

Placental inflammatory response (PIR) is associated with adverse neonatal outcomes such as sepsis, cerebral palsy, low birth weight, preterm birth, and neonatal mortality. However, there is an urgent need for noninvasive and sensitive biomarkers for prediction of PIR. In this study, we evaluated the clinical usefulness of maternal serum inflammatory markers for prediction of PIR in women with impending preterm birth. We conducted a retrospective cohort study of 483 patients who delivered preterm neonates. Serum levels of leukocyte differential counts, C-reactive protein (CRP), and neutrophil to lymphocyte ratio (NLR) were compared between women with no placental inflammation and women with PIR. The mean neutrophil counts, CRP levels, and NLR in both the patients with histologic chorioamnionitis (HCA) alone and those with HCA with funisitis were significantly higher than those in women with no placental inflammation. Compared to leukocyte subset or CRP, NLR in women with funisitis was significantly higher than in women with HCA alone and showed higher predictive accuracy, along with 71.4% sensitivity, 77.9% specificity, 80.7% positive predictive value, and 67.8% negative predictive value for prediction of PIR. On Kaplan-Meier survival analysis, women with both an elevated level of CRP and a high NLR had a shorter admission-to-delivery interval compared to women with either an elevated level of CRP or a high NLR alone. NLR may be a predictive marker of PIR and could be used as a cost-effective parameter for identifying women at risk of PIR.

## Introduction

Intrauterine inflammation is thought to be the principal contributor to the onset of preterm parturition [1], [2]. The inflammatory responses of the placenta and umbilical cord, as markers of intrauterine inflammation, have been classified as maternal inflammatory response (MIR) and fetal inflammatory response (FIR). While MIR occurs as inflammation infiltrates into the chorion, amnion, or decidua, FIR occurs when the inflammation is extended to the chorionic plate, umbilical cord, and fetal vessels themselves and is defined as a fetal plasma Interleukin-6 concentration >11 pg/mL [3]–[5].

Histologic chorioamnionitis (HCA) is regarded as a hallmark of maternal inflammation of the placenta, whereas funisitis, or inflammation of the umbilical cord, is a marker of fetal inflammation [4]. HCA is often present in the placentas of patients who experience preterm labor or preterm premature rupture of membranes (pPROM) [6]. Preterm infants from mothers with HCA are at high risk of developing adverse neonatal outcomes. Numerous published studies have addressed the impact of HCA on neonatal sepsis, intraventricular hemorrhage, cerebral white matter abnormalities, bronchopulmonary dysplasia, cerebral palsy, and neonatal mortality [7]–[9]. Funisitis often represents FIR; the presence of funisitis is related to a more advanced state of intrauterine inflammation. FIR with extensive placental involvement displays a wide spectrum of severity, and the additional presence of FIR appears to exacerbate the detrimental effects of chorioamnionitis on neonatal outcomes [5], [10].

Therefore, prenatal diagnosis of placental inflammatory response (PIR) is of great clinical importance in providing information that may be useful in determining the prognosis and treatment strategy for a pregnant woman and neonate at risk of preterm birth. In this study, a noninvasive and rapid prenatal HCA diagnostic method using a maternal serum marker was developed, and the usefulness of the method as a predictive factor for intrauterine inflammation was evaluated.

## Materials and Methods

This retrospective study included data from 483 consecutive patients who delivered preterm neonates at the Department of Obstetrics and Gynecology, Gangnam Severance Hospital, Yonsei University College of Medicine, between June 2007 and May 2013. We included patients who met the following criteria: (1) singleton gestation, (2) Preterm delivery occurring between 24 and 37 weeks of gestation (3) spontaneous preterm births as a result of preterm labor or pPROM (4) results available for histopathologic examination of the placenta, (5) no history of cervical cerclage, (6) no major congenital anomalies or intrauterine fetal death, and (7) the absence of preexisting maternal or placental diseases. All study participants signed a written informed consent and the protocol of this study was approved by the Institutional Review Board of Gangnam Severance Hospital (IRB No. 3-2013-0131).

The clinical characteristics of the mothers and infants were retrieved from a review of medical records. Maternal blood was collected at the time of admission, prior to administration of antibiotics, corticosteroids, or tocolytics. For all study subjects, serum levels of leukocyte differential counts and C-reactive protein (CRP) obtained at admission were collected retrospectively. The neutrophil to lymphocyte ratio (NLR) was defined as the absolute neutrophil count divided by the absolute lymphocyte count. Additionally, vaginal swabs were taken from all study subjects for detection of aerobic and anaerobic bacterial overgrowth, *Candida* colonization, *Ureaplasma urealyticum* and *Mycoplasma hominis* colonization at the time of admission. We also analyzed the pregnancy outcomes of all study subjects to evaluate the clinical significance of maternal inflammatory markers as a prognostic marker. We defined pregnancy outcome as the time interval from admission to delivery.

Histologic data were obtained by reviewing placental pathology reports. In all cases, histologic examination of the placenta, umbilical cord, and fetal membranes was performed. Paraffin-embedded tissue blocks were sectioned and stained with hematoxylin and eosin (H&E). Placental inflammatory status was classified based upon the following criteria: MIR was defined as subchorionitis, chorioamnionitis, deciduitis, or free membranitis without funisitis or chorionic plate vasculitis; FIR was defined as inflammation extending to the fetal side of the placental unit (funisitis or chorionic plate vasculitis). Only four women with FIR in the absence of MIR were excluded from the subsequent statistical analyses.

We performed the Shapiro-Wilk test for testing the normality of data. Clinical characteristics and pregnancy outcomes were compared using non-parametric test for continuous variables and presented as the mean ± standard deviation or mean (range). Multiple comparisons among three groups were conducted by Kruskal-Wallis test and differences between two groups were assessed by Mann-Whitney U test with Bonferroni correction (p<0.0167). Categorical variables were compared using Chi-square test or Fisher’s exact test and presented as numbers (%). Laboratory data was assessed by analysis of covariance (ANCOVA) with gestational age as the covariate.

Receiver operating characteristic (ROC) analysis was used to estimate the specificity and sensitivity of each marker. We calculated the area under the curve (AUC), which indicates the average sensitivity of a marker over the entire ROC curve, and determined the optimal cut-off value resulting in the highest sum of sensitivity and specificity for each marker. Survival plots were drawn using the Kaplan-Meier method to evaluate the relationship between pregnancy outcome and the result of each marker, and the differences were calculated using the log-rank test. Univariate and multivariate logistic regression analysis were performed to evaluate independent prognostic factors associated with PIR. For all analyses, a P<0.05 was considered statistically significant except pair-wise comparison. Statistical analyses were performed using SPSS version 17.0 (SPSS Inc., Chicago, IL).

## Results

### Clinical characteristics of the study population

The study population consisted of 483 patients who delivered preterm neonates between June 2007 and May 2013. On placental histologic examination of the study subjects, 59.4% (287/483) had evidence of PIR. Among the 287 women with PIR, HCA alone was observed in 85.7% (246/287), and combined HCA and funisitis was identified in 14.3% (41/287). Table 1 shows the clinical characteristics and pregnancy outcomes of the study subjects according to the presence or absence of HCA and funisitis. There were significant differences in antenatal corticosteroid use, gestational age at hospitalization and delivery, neonatal intensive care unit admission, APGAR score at 1 and 5 minutes, and birth weight among the three groups. While MIR and FIR were significantly more frequent in early preterm and moderately preterm births, normal placenta was more frequent in late preterm birth. These results showed that MIR and FIR are more common placental pathological findings in the early preterm and moderately preterm births and its frequency decreases with advancing gestation.

**Table 1:** Clinical characteristics and pregnancy outcomes of study subjects.

	Placental inflammatory response status						
	None (n = 196)	*P* a	MIR (n = 246)	*P* b	FIR (n = 41)	*P* c	*P* d
**Clinical characteristics**							
Maternal age (years)	31.9±3.6	0.973	31.6±3.8	0.856	31.7±3.1	0.850	0.981
BMI (kg/m^2^)	25.1±3.5	0.109	24.6±3.9	0.260	25.8±4.5	0.848	0.209
Gravidity	2.1±1.3	0.591	2.2±1.4	0.498	1.9±1.1	0.711	0.739
Parity	0.5±0.7	0.761	0.5±0.7	0.576	0.5±0.7	0.699	0.842
Number of abortions	0.6±1.0	0.555	0.7±1.1	0.477	0.5±0.7	0.716	0.709
Previous preterm birth history	10 (5.1)	0.653	15 (6.1)	0.729	3 (7.3)	0.476	0.824
Preterm labor	103 (52.6)	0.537	122 (49.6)	0.847	20 (48.8)	0.877	0.826
pPROM	93 (47.4)	0.537	124 (50.4)	0.923	21 (51.2)	0.66	0.799
Active labor*	19 (9.7)	0.185	34 (13.8)	>0.999	5 (12.2)	0.578	0.415
Cervical dilatation at admission (cm)	1.2±1.8	0.141	1.5±2.1	0.809	1.6±2.3	0.538	0.334
Antenatal corticosteroid use	**97 (49.5)**	**<0.001**	**81 (32.9)**	**0.004**	**23 (56.1)**	0.442	**<0.001**
Antibiotics use	114 (58.2)	0.858	141 (57.3)	0.466	21 (51.2)	0.414	0.714
Tocolytics use	89 (45.4)	0.127	94 (38.2)	0.2	20 (48.8)	0.694	0.206
**Pregnancy outcomes**							
Cesarean delivery	103 (52.6)	0.225	115 (46.7)	0.412	22 (53.7)	0.897	0.416
Gestational age at hospitalization (weeks)	**32.7 (24.3–36.4)**	**<0.001**	**30.2 (24.1–36.9)**	0.201	**29.5 (24.0–36.7)**	**<0.001**	**<0.001**
Gestational age at delivery (weeks)	**33.4 (24.4–36.6)**	**<0.001**	**30.5 (24.1–36.9)**	0.394	**30.0 (24.0–36.7)**	**<0.001**	**<0.001**
Early preterm birth (<28 weeks)	**13 (6.6)**	**<0.001**	**73 (29.7)**	0.484	**13 (31.7)**	**<0.001**	**<0.001**
Moderately preterm birth (29–33 weeks)	**72 (36.7)**		**115 (46.7)**		**21 (51.2)**		
Late preterm birth (34–36 weeks)	**111 (56.6)**		**58 (23.6)**		**7 (17.1)**		
Admission to delivery interval (days)	4.6±10.8	0.305	2.4±7.7	0.850	3.7±10.2	0.737	0.594
Neonatal intensive care unit admission	**112 (57.1)**	**0.002**	**175 (71.4)**	0.061	**35 (85.4)**	**0.001**	**<0.001**
APGAR score at 1 min	**5.7±1.8**	**<0.001**	**4.1±2.7**	0.797	**4.1±2.5**	**<0.001**	**<0.001**
APGAR score at 5 min	**7.2±1.7**	**<0.001**	**5.5±3.1**	0.732	**5.7±2.6**	**<0.001**	**<0.001**
Birth weight (g)	**1996.0±731.6**	**<0.001**	**1655.6±1004.3**	0.795	**1602.3±991.3**	**0.013**	**<0.001**

Values are given as mean ± standard deviation, n (%) or median (range).

MIR, maternal inflammatory response; FIR, fetal inflammatory response; BMI, body mass index; pPROM, preterm premature rupture of membranes.

*Active labor was defined as cervical dilatation of 3 cm or more in the presence of regular uterine contractions.

a Comparison between groups with no placental inflammation and MIR;

b Comparison between groups with MIR and FIR;

c Comparison between groups with no placental inflammation and FIR;

d Comparison among groups with no placental inflammation, MIR and FIR.

### Leukocyte differential counts, CRP, NLR, and vaginal cultures

Leukocyte differential counts, CRP levels, NLR, and the results of vaginal cultures are shown in Table 2. There were significant differences in the mean neutrophil counts, mean lymphocyte counts, and serum CRP levels among women with no placental inflammation, HCA alone, and HCA with funisitis. The mean neutrophil counts and CRP levels in both the patients with HCA alone and those with HCA with funisitis were significantly higher than those in women without any placental inflammation. The lymphocyte counts in both the patients with HCA alone and those with HCA with funisitis were significantly lower than those in women without any placental inflammation. Because the neutrophil and lymphocyte counts in all HCA groups were significantly different from those of women without HCA, we investigated the diagnostic and prognostic significance of NLR. The NLR was significantly higher in both women with HCA alone and in those with HCA with funisitis than in women without any placental inflammation, and differences could be distinguished among all three groups; the NLR in women with funisitis was significantly higher than that in women with HCA alone. With regard to CRP levels, however, there was no difference between these two groups. In addition, there were no differences in the results of vaginal cultures, including *U. urealyticum* and *M. hominis*, among the three groups.

**Table 2:** Mean values of leukocyte differential counts, CRP and NLR in study subjects.

	Placental inflammatory response status							
	None (n = 196)		*P* a	MIR (n = 246)	*P* b	FIR (n = 41)	*P* c	*P* d
**Serum inflammatory markers**								
Neutrophil (cells/µl)	7987.6±3073.0	<0.001		10503.5±4686.5	0.214	11473.7±3754.2	<0.001	<0.001
Lymphocyte (cells/µl)	1682.4±813.8	<0.001		1394.3±557.7	0.46	1307.3±610.0	0.003	<0.001
Monocyte (cells/µl)	437.4±165.7	0.024		475.5±187.9	0.374	459.3±157.9	0.649	0.073
Eosinophil (cells/µl)	102.1±75.7	0.839		109.5±110.0	0.303	95.9±61.4	0.274	0.536
Basophil (cells/µl)	31.5±21.8	0.465		33.5±25.4	0.968	33.7±22.3	0.646	0.75
CRP (mg/L)	7.1±16.7	0.006		16.8±23.4	0.157	23.5±38.9	0.003	0.002
NLR	5.4±3.5	<0.001		8.8±5.6	0.004	11.6±9.4	<0.001	<0.001
**Vaginal cultures**								
*Ureaplasma urealyticum*	88(44.9)		0.698	115(46.7)	0.735	18(43.9)	0.907	0.899
*Mycoplasma hominis*	5(2.6)		0.226	12(4.9)	0.701	1(2.4)	>0.999	0.396
Other bacteria	58(29.6)		0.941	72(29.3)	>0.999	12(29.3)	>0.999	0.997

Values are given as mean ± standard deviation or n (%) unless otherwise specified.

MIR, maternal inflammatory response; FIR, fetal inflammatory response; CRP, C-reactive protein; NLR, neutrophil to lymphocyte ratio.

a Comparison between groups with no placental inflammation and MIR;

b Comparison between groups with MIR and FIR;

c Comparison between groups with no placental inflammation and FIR;

d Comparison among groups with no placental inflammation, MIR and FIR.

### Diagnostic significance of NLR in predicting PIR

**Figure 1:**
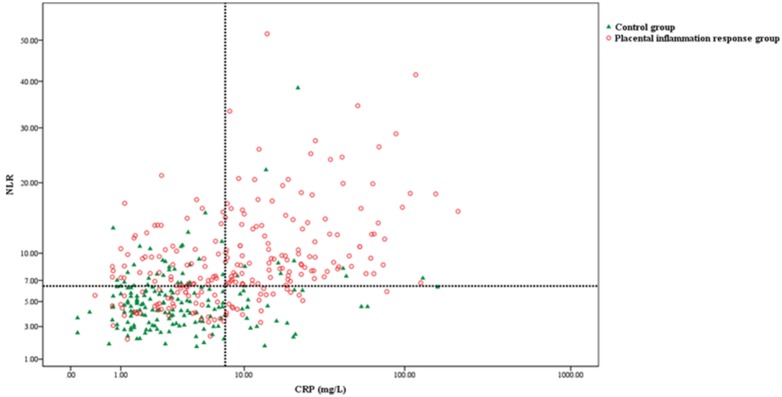
Comparison of neutrophil to lymphocyte ratio (NLR) (y-axis) and C-reactive protein (CRP) (x-axis) in placental inflammatory response group (red open circle) and control group (green filled triangle). The dotted lines indicate the optimal cut-off values (6.48) of NLR and CRP (7.46) which maximize the sum of sensitivity and specificity.

Using ROC curve analysis, we compared the diagnostic indices and predictive values of leukocyte differential counts, CRP levels, and the NLR in predicting PIR (Table 3). The NLR had the highest AUC value of 0.798 (95% CI, 0.756–0.841) with a cut-off value of 6.48, and it had a sensitivity of 71.4%, specificity of 77.9%, positive predictive value (PPV) of 80.7%, and negative predictive value (NPV) of 67.8% as a predictor for PIR. For CRP levels, the AUC was 0.727 (95% CI, 0.679–0.776) with a cut-off value of 7.46, along with 56.8% sensitivity, 82.9% specificity, 81.1% PPV, and 59.8% NPV. To categorize study subjects as CRP and NLR positive or negative, an optimal cut-off value was chosen to maximize the sum of sensitivity and specificity in the ROC curve. We also assessed the effectiveness of the NLR as an additional diagnostic marker to CRP levels for predicting PIR. In women with PIR (n = 287), CRP-negative (<7.46 mg/L) status was found in 124 (43.2%) of 287 patients. Of 124 patients with a false negative in CRP levels, 65 (52.4%) were NLR positive (≥6.48), which could be used as an additional diagnostic marker of PIR (Figure 1).

**Table 3:** Diagnostic indices of leukocyte differential counts, CRP and NLR in study subjects.

	AUC (95% CI)	Sensitivity (%)	Specificity (%)	PPV (%)	NPV (%)	Cutoff Value
Neutrophil	0.706 (0.656–0.755)	49.6	85.6	81.7	56.8	10865
Lymphocyte	0.348 (0.296–0.401)	49.6	28.2	47.2	30.2	1235
Monocyte	0.541 (0.485–0.597)	72.2	36.5	59.5	50.4	365
Basophil	0.488 (0.432–0.544)	8.5	94.5	66.7	44.4	65
Eosinophil	0.473 (0.417–0.529)	3.4	99.4	88.9	44.3	350
CRP	0.727 (0.679–0.776)	56.8	82.9	81.1	59.8	7.46
NLR	0.798 (0.756–0.841)	71.4	77.9	80.7	67.8	6.48

AUC, area under the curve; CI, confidence interval; PPV, positive predictive value; NPV, negative predictive value; CRP, C-reactive protein; NLR, neutrophil to lymphocyte ratio.

### Prognostic significance of NLR in pregnancy outcome

**Figure 2:**
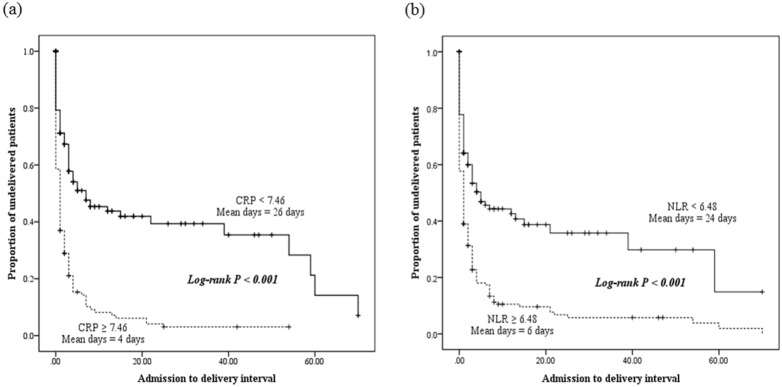
Kaplan-Meier overall survival of admission-to-delivery intervals according to C-reactive protein (CRP) (a) and neutrophil to lymphocyte ratio (NLR) (b). CRP-positive (≥7.46), NLR-positive (≥6.48), dotted line; CRP-negative (<7.46), NLR-negative (<6.48), broken line.

**Figure 3:**
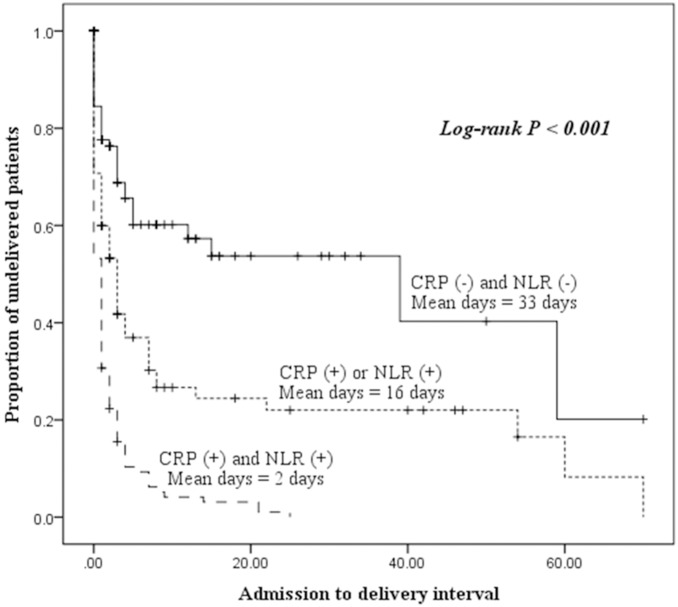
Kaplan-Meier overall survival of admission-to-delivery intervals according to C-reactive protein (CRP) levels and neutrophil to lymphocyte (NLR) status.

Figure 2 shows the Kaplan-Meier survival analysis for CRP levels and the NLR. It reveals that CRP-positive (≥7.46 mg/L) women had a significantly shorter admission-to-delivery interval than CRP-negative (<7.46 mg/L) women (median, 4 vs. 26 days; log rank, P<0.001), and NLR-positive (≥6.48) women had a significantly shorter admission-to-delivery interval compared to NLR-negative (<6.48) women (median, 6 vs. 24 days; log rank, P<0.001). When the patients were classified into three groups based on CRP levels and the NLR, patients with both an elevated level of CRP and a high NLR had a shorter admission-to-delivery interval compared to patients with either an elevated level of CRP or a high NLR (Figure 3).

### Relationship between clinical and laboratory parameters and PIR

To assess the relative importance of demographic and clinical characteristics in the prediction of PIR, we performed multiple logistic regression analysis with variables to be considered risk factors for PIR and clinical parameters (Table 4). Using the cut-off values derived from the ROC curves, elevated levels of CRP (≥7.46 mg/L) and NLR (≥6.48) were significantly associated with PIR, even after controlling for gestational age at hospitalization and delivery, monocytes, and ureaplasma infection.

**Table 4:** Adjusted risk factors for PIR in patients with preterm delivery.

Risk factor	Adjusted odds ratio (95% CI)	*P*
Gestational age at admission		
<28	1.72 (0.31–9.48)	0.532
28–32	0.52 (0.17–1.58)	0.248
>33	1 (referent)	
Gestational age at delivery		
<28	2.85 (0.46–17.52)	0.259
28–32	2.77 (0.89–8.61)	0.078
>33	1 (referent)	
CRP>7.46	3.40 (1.83–6.34)	<0.001
NLR>6.48	5.18 (2.95–9.10)	<0.001

PIR, placental inflammatory response; CI, confidence interval; CRP, C-reactive protein; NLR, neutrophil to lymphocyte ratio.

## Discussion

The principal findings of this study were as follows: 1) the NLR had a better overall diagnostic performance than maternal serum CRP levels in predicting PIR and distinguishing HCA from funisitis; 2) patients with a high NLR were at risk of impending preterm delivery in the context of normal CRP levels; and 3) the combined use of NLR and CRP levels measured before treatments can predict poor pregnancy outcomes in patients with PIR.

In general, amniotic fluid analysis and histologic examination of the placenta are important methods of diagnosing intrauterine inflammation. The examination includes gram staining of the amniotic fluid and measurement of leukoattractants, glucose concentration, white blood cell count, and interleukin 6 (IL-6) [11], [12]. However, an important limitation of these examination methods is that amniocentesis, an invasive procedure, is unavoidable. In addition, diagnosis of HCA through histologic examination of the placenta is possible only after delivery; thus, the method is not suited for rapid prenatal diagnosis of intrauterine inflammation, nor can it be used in neonatal treatment in patients diagnosed with neonatal sepsis. Therefore, many researchers have attempted to develop a prenatal, noninvasive, and rapid method of diagnosing intrauterine inflammation. Noninvasive diagnostic tools used to assess the risk of HCA include measurement of maternal serum CRP levels and leukocyte counts, but these conventional infection markers, when used alone, have poor diagnostic value in distinguishing patients with HCA from those with no placental inflammation.

Because the physiological immune response of circulating leukocytes to systemic inflammation is accompanied by increased neutrophils and decreased lymphocytes, the NLR has been proposed as a simple parameter of systemic inflammation and stress in various diseases [13]–[15]. Earlier, we had already shown that a combined marker, including the NLR, provided a more sensitive parameter than the cervix length alone in the prediction of preterm birth [16]. Given that HCA has been shown to be associated with increased blood levels of inflammatory markers, we hypothesized that the NLR should be affected during the inflammatory processes of HCA.

The present study showed that an increased NLR is an appropriate indicator for prenatal diagnosis of HCA. According to the ROC curve analysis, maternal NLR at the time of admission has greater diagnostic usefulness than CRP level with regard to acute HCA and may serve as a useful marker to distinguish HCA with funisitis from HCA without funisitis. Our results showed that intrauterine inflammation may be prenatally predicted even without the use of an invasive diagnostic method such as amniocentesis. Moreover, we investigated whether combined use of CRP level and NLR, measured before treatment, can predict poor pregnancy outcomes in patients with PIR. We observed significant prognostic differences among patients having both a normal CRP level and a low NLR, those with either an elevated level of CRP or a high NLR, and those with both an elevated level of CRP and a high NLR. These results indicate that the combination of CRP level and NLR may help to improve prognostic accuracy in patients with PIR. Although the NLR is limited in providing all necessary information as a predictive marker, it can still complement other inflammatory markers in predicting HCA. In addition, a mother experiencing preterm labor in whom the NLR is increased at the time of admission may have a high possibility of preterm birth due to the failure of suppression of uterine contraction, and she may subsequently have a poor pregnancy outcome, with delivery taking place in a shorter time interval from admission compared to a pregnant woman in whom the NLR is not high. Our multivariable logistic regression analysis showed that the NLR may independently predict PIR, without being affected by other factors, providing evidence that it is a very useful test in predicting the prognosis of pregnancy.

It was assumed that the cytokines and chemokines secreted from the partial inflammatory lesion in the choriodecidua generated during the early stage of intrauterine inflammation may enter the blood of a pregnant woman and cause changes in the counts of leukocyte subtypes [17], [18]. A strong host immune response that results from an increase in the local production of proinflammatory cytokines and chemokines such as IL-1β, IL-6, IL-10, tumor necrosis factor-α (TNF-α), granulocyte colony-stimulating factor (G-CSF), prostaglandins, and leukotrienes eventually allows neutrophilia to occur [19]. Lymphopenia, on the other hand, results from inflammation-induced mechanisms such as impaired antigen presentation, activated negative costimulatory signals, and production of immunosuppressive factors, all of which can contribute to a significant decrease in T-helper lymphocytes [20]. In the early phase of the inflammatory response, the TNF family members are induced, thus increasing both the expression of receptors on lymphocytes and also lymphocyte apoptosis. This phenomenon occurs primarily because the protective lymphocyte-dependent immune responses are reduced by marked decreases in lymphocytes [21], [22].

Numerous studies have attempted to identify useful biomarkers for HCA through the analyses of amniotic fluid and maternal blood [11], [23]–[25]. CRP, produced by the liver as an acute phase protein, is a nonspecific marker generated in response to inflammatory stimuli. As one of many major laboratory tests, CRP is primarily used when an obstetrician needs to monitor various inflammatory conditions such as chorioamnionitis [24]. However, according to recent systemic reviews and a meta-analysis conducted by Lamont's group, maternal serum CRP may not be considered a reliable indicator of either clinical or histologic chorioamnionitis due to the presence of differences among studies [26]. In addition, Romero et al. [6] reported correlations among the existence of a placental inflammatory lesion, inflammation level, and choriodecidua inflammation pattern via amniotic fluid culture results and amniotic fluid white blood cell counts, respectively. Although HCA and amniotic fluid culture tests are useful in diagnosing intrauterine inflammation and predicting the prognosis of a neonate, they have drawbacks, such as the time needed for culture and the high possibility of a false negative result due to various causes. Furthermore, Romero et al. [27] and Yoon et al. [11] reported that IL-6 concentration in amniotic fluid is a more sensitive measure for predicting intrauterine inflammation than gram staining, the glucose concentration, or the white blood cell count of amniotic fluid. Among various cytokines, many studies in the past investigated IL-6, as it could be measured relatively easily. However, with recent developments in measurement methods, various cytokines such as IL-1β, IL-8, TNF-α, matrix metalloproteinase-8, 9 (MMP-8, 9), and IL-1 receptor agonist are now being further measured. The method of measuring the cytokine concentration in amniotic fluid appears very promising, having achieved particularly high sensitivity and specificity. However, this method is not widely performed because it is relatively difficult in practice and the cost is high.

Importantly, in our study, the NLR performed significantly better than either CRP level or neutrophil counts in the prediction of PIR, although these last two markers are used widely in the diagnosis of HCA. As demonstrated in this study and described in our previous studies, maternal blood NLR may be considered the most useful, noninvasive prenatal diagnostic method currently known. Measuring the NLR in a pregnant woman experiencing preterm labor or pPROM may reduce the frequency of unnecessary amniocentesis to as low a level as possible. Moreover, measuring the NLR has other advantages, such as that the test sample may be simply obtained and the NLR may be easily measured using conventional facilities, without the need of additional instruments or reagents.

This study is the first to ever examine and report an independent diagnostic and prognostic value of measuring maternal serum NLR for HCA and funisitis. The limitation of this study, however, is its retrospective design and the results here should, ideally, be confirmed by subsequent larger prospective studies. Furthermore, since we used a single blood sample to calculate NLR, it is uncertain whether a single sample could reflect an elevated NLR over time.

In conclusion, however, the significance of this study is that it showed that a placental inflammatory change may be simply and quickly verified at low expense by measuring the NLR. Further studies may still be needed to explore whether injecting an antibiotic in a pregnant woman with an increased NLR may reduce various neonatal complications and various sequelae of neonates accompanying histologic chorioamnionitis.
